# Metabolite Profile of Xylem Sap in Cotton Seedlings Is Changed by K Deficiency

**DOI:** 10.3389/fpls.2020.592591

**Published:** 2020-12-10

**Authors:** Xin Zhang, Guo Wang, Huiyun Xue, Jinbao Zhang, Qinglian Wang, Zhiyong Zhang, Baohong Zhang

**Affiliations:** ^1^Henan Collaborative Innovation Centre of Modern Biological Breeding, Henan Institute of Science and Technology, Xinxiang, China; ^2^Department of Biology, East Carolina University, Greenville, NC, United States

**Keywords:** metabolite, cotton, xylem sap, potassium, deficiency

## Abstract

Xylem sap, belonging to the plant apoplast, not only provides plant tissues with inorganic and organic substances but also facilitates communication between the roots and the leaves and coordinates their development. This study investigated the effects of potassium (K) deficiency on the morphology and the physiology of cotton seedlings as well as pH, mineral nutrient contents, and metabolites of xylem sap. In particular, we compared changes in root–shoot communication under low K (LK) and normal K (NK, control) levels. Compared to control, LK stress significantly decreased seedling biomass (leaf, stem, and root dry weight; stem and root length; root surface area and root volume) and the levels of K, Na (sodium), Mg (magnesium), Fe (iron), and Zn (zinc) in xylem sap. A total of 82 metabolites in sap analyzed by high-performance liquid chromatography–tandem mass spectrometry (HPLC–MS/MS) showed significant differences between the two conditions; among these, 38 were up-regulated more than 2-fold, while the others were down-regulated less than 0.5-fold. In particular, several metabolites found in the cell membrane including three cholines (glycerophosphatecholine, 2-hexenylcholine, and caproylcholine) and desglucocoroloside and others such as malondialdehyde, α-amino acids and derivatives, sucrose, and sugar alcohol significantly increased under LK stress, indicating that cell membranes were damaged and protein metabolism was abnormal. It is worth noting that glycerophosphocholine was up-regulated 29-fold under LK stress, indicating that it can be used as an important signal of root–shoot communication. Furthermore, in pathway analyses, 26 metabolites were matched to Kyoto Encyclopedia of Genes and Genomes (KEGG) pathways; L-aspartic acid, which was associated with 10 KEGG pathways, was the most involved metabolite. Overall, K deficiency reduced the antioxidant capacity of cotton seedlings and led to a metabolic disorder including elevated levels of primary metabolites and inhibited production of secondary metabolites. This eventually resulted in decreased biomass of cotton seedlings under LK stress. This study lays a solid foundation for further research on targeted metabolites and signal substances in the xylem sap of cotton plants exposed to K deficiency.

## Highlights

- Elaborated the morphology, physiological characteristics, and metabolites of xylem sap altered by potassium (K) deficiency in cotton.
- First report of the metabolome changes of xylem sap in cotton seedlings under K deficiency.
- K deficiency increased the contents of primary metabolites and inhibited the production of secondary metabolites.

## Introduction

Potassium (K) is a macronutrient, and unlike nitrate, phosphate, and sulfate, it is not assimilated into organic matter. It rather plays important roles in plant cells (e.g., expansion, turgor pressure, and osmo-regulation) and is involved in metabolism, growth, yield, and the opening and the closing of stomata in response to abiotic and biotic stresses (Hasanuzzaman et al., 2018; Chérel and Gaillard, 2019). K is involved directly or indirectly in plant protein and sugar metabolism (Chen et al., 2018; Andrea et al., 2020) and is required by more than 60 enzymes as a cofactor (Hawkesford et al., 2012; Vašák and Schnabl, 2016). Its levels affect those of primary and secondary metabolites in plants (Armengaud et al., 2009; Coskun et al., 2017; Chatterjee et al., 2020).

K deficiency is a common abiotic stress in agricultural production (Hosseini et al., 2017; Xu et al., 2020). It can lead to increases in the concentrations of free sugars in the leaves of bean (Cakmak et al., 1994), cotton (Bednarz and Oosterhuis, 1999; Pettigrew, 1999; Hu et al., 2017), potato (Koch et al., 2018), and oilseed rape (Pan et al., 2017), the roots of alfalfa (Jungers et al., 2019), rice (Ma et al., 2012; Chen et al., 2015), and sugar beet (Aksu and Altay, 2020), and the leaves and the roots of *Arabidopsis* (Armengaud et al., 2009), barley (Zeng et al., 2018), and tomato (Sung et al., 2015). In addition, excessive accumulation of free amino acids, especially proline, has been reported for tobacco (Ren et al., 2016), cotton (Hu et al., 2017), oilseed rape (Lu et al., 2019), barley (Zeng et al., 2018), and *Arabidopsis* (Armengaud et al., 2009). Significant changes in the metabolite profile of plants induced by K deficiency can lead to metabolism disorders (Hu et al., 2016).

Metabolomics (also known as metabonomics or metabolic profiling) is an emerging branch of “omics” research concerned with the comprehensive identification and quantification of small metabolites (molecular weight < 1 kDa) in organisms. Metabolites are the downstream products of numerous proteome-wide interactions, and non-targeted quantitative analyses of such metabolites in bio-fluids and tissues can be a very sensitive measure of an organism’s phenotype (Hong et al., 2016). Hence, metabolomics can be particularly useful for identifying metabolic components contributing the most to key phenotypic and physiological traits (Thomason et al., 2018), studying environment–gene interactions (Ronny et al., 2013), and identifying disease or stress biomarkers (Peng et al., 2015).

Liquid chromatography–electrospray ionization–mass spectrometry (LC–ESI–MS) is a highly sensitive technique that provides information on the molecular masses of compounds. To obtain further structural information on the fragmentation patterns of compounds, ESI-tandem mass spectrometry (MS/MS) can be used (Mari et al., 2015; Prabakaran et al., 2018). LC–MS-based metabolomics approaches are widely used to profile complex biological extracts such as those from plants (Shimizu et al., 2018; Feussner and Feussner, 2019), being of particular importance for non-targeted plant metabolomics, mainly due to the plant kingdoms’ rich metabolite diversity (Gorrochategui et al., 2016; Wang et al., 2019). Compared to other analytical techniques, the main advantages of LC–MS are that it can cover a wide chemical diversity and a wide dynamic range; it is a complementary analytical technique to nuclear magnetic resonance (NMR) and gas chromatography–MS in metabolomics studies (Anderson et al., 2020; Yaglioglu et al., 2020). The traditional untargeted LC–MS device consists of an LC with a reverse-phase chromatography (RPC) column (with an inner diameter of 2.1 or 4.6 mm) for analyses in front of a mass spectrometer. Although the separation range of RPC columns is quite wide, they cannot retain small polar metabolites well. Hydrophilic interaction liquid chromatographic (HILIC) columns are increasingly used for analyses of polar compounds (Wolf et al., 2012; Chai et al., 2018; Taraji et al., 2018), although they have a lower loading capacity than RPC columns, which results in wider peak shapes (Lurie et al., 2011).

The plant apoplast, which includes xylem sap, serves as an interface between the environment and the protoplast. It is not only a barrier against adverse stresses but also has multiple functions in metabolism and signal transduction (Zhang et al., 2008). Several studies have characterized plant defenses against biotic (Feussner and Feussner, 2019; Green et al., 2020) and abiotic (Hasanuzzaman et al., 2018; Chérel and Gaillard, 2019; Lu et al., 2019) stresses by analyzing the metabolites of the apoplast. The metabolites in xylem sap secreted from the root symplast not only reflect the physiological state of roots but also affect the growth and the development of above-ground plant parts (Zhang Z. et al., 2016). For cotton, K deficiency is a worldwide problem (Zhao et al., 2014). K deficiency leads to an imbalance of the phytohormone abscisic acid and cytokines in the xylem sap (Wang et al., 2012) and induces premature leaf senescence (Li et al., 2012; Hu et al., 2016). However, to the best of our knowledge, no previous study has investigated the response mechanism of cotton to K deficiency by analyzing the metabolic components of xylem sap.

## Materials and Methods

### Cultivar and Culturing Conditions

Cotton cultivar “DP 99B” was used in this experiment. A randomized block design was employed, with eight plants per pot and 12 pots per treatment. Cotton seedlings were cultivated in a culture room of the Henan Institute of Science and Technology under a regime of 14-h light/10-h dark, with a temperature of 30 ± 2°C during the day and at 25 ± 2°C at night.

### Culture of Seedlings

Seeds of similar size were selected and sterilized with 9% H_2_O_2_ for 30 min. After having been rinsed with tap water, the seeds were planted in wet sand. As soon as the seedlings had reached the stage where two cotyledons were expanding from the seed case, they were transferred to containers with a nutrient solution with normal K (NK) concentration. After culturing for 3 days, the seedlings were again transferred to containers with normal or low K concentration to grow for a further 7 days; during this time, samples of xylem sap, roots, and leaves were collected.

The nutrient solution was continuously aerated and contained 2.5 mmol L^–1^ Ca(NO_3_)_2_, 1 mmol L^–1^ MgSO_4_, 0.5 mmol L^–1^ NH_4_H_2_PO_4_, 2 × 10^–4^ mmol L^–1^ CuSO_4_, 1 × 10^–3^ mmol L^–1^ ZnSO_4_, 0.1 mmol L^–1^ EDTAFeNa, 2 × 10^–2^ mmol L^–1^ H_3_BO_3_, 5 × 10^–6^ mmol L^–1^ (NH_4_)_6_Mo_7_O_24_, and 1 × 10^–3^ mmol L^–1^ MnSO_4_. K was added in the form of KCl, with a final concentration of 0.05 mmol L^–1^ in the LK culture solution and 2.5 mmol L^–1^ in the NK culture solution.

### Determining the Morphological Indexes of Cotton Seedlings

An EPSON 12000XL scanner was utilized to scan the root, stem, and leaf, respectively, spread out on a transparent plastic tray after being separated from the cotton seedlings. Some water was usually added to the tray to benefit root spread. WinRHIZO Pro 2017 software was used to examine their stem length, total root length, root surface area, mean diameter of root, and root volume. The scanned seedling parts were weighed as fresh weight after water was allowed to drip for the roots to dry. Finally, fresh seedlings were placed in paper bags, baked at 105°C for 20 min, and then dried at 80°C to a constant weight for weighing.

### Xylem Sap Collection and Preparation

Xylem sap was collected *via* natural root pressure. In details, firstly, the up-ground part of a seedling was cut approximately 5 cm above the junction of the root and the stem; secondly, after washing the rootstock surface five times with distilled water to clean possible substances of pollution from phloem, the xylem sap was blotted with filter paper, then a latex tube was fitted over 0.5 cm rootstock, and the other end of the tube was put into a plastic centrifuge tube of 15 ml, which was placed in a foam box filled with ice. The sap of six to eight seedlings was collected into each plastic tube. The samples were frozen with liquid nitrogen and then kept at −80°C for subsequent testing.

When exerting further experiments, the frozen collected sap was thawed and first coarsely filtered through a 0.25 μm inorganic filter membrane; the filtered xylem sap was transferred into a 3-KD ultra-centrifuge tube (Millipore’s Amicon Ultra-4) to separate substances with molecular weight >3 KD and <3 KD. The samples were centrifuged at 7,500 *g* for 45 min at 4°C to obtain a concentrated protein solution in the smaller and inner centrifuge tube and permeated metabolite and ion liquid in the bigger and outer centrifuge tube. The former was used to determine the free protein content and activity of peroxidase (POD) and superoxide dismutase (SOD) enzymes, and the latter was used to determine free sugar, amino acids, polyphenols, and the metabolite profile.

### Determination of Physiological Indicators of Xylem Sap

#### Determination of pH Value and Cation Contents

The pH value was measured using a pH meter. The levels of K, Na (sodium), Ca (calcium), Mg (magnesium), Fe (iron), and Zn (zinc) in the xylem sap was assessed *via* inductively coupled plasma luminescence spectrometry after diluting the sap with 2% HCl, at a sap/HCl ratio of 1:100, and then oscillating the samples at a speed of 100 *g* for 10 min.

#### Determination of Physiological Indexes Related to Stress Resistance

The content of free protein was determined using the Coomassie brilliant blue method (Bradford, 1976), and the activities of POD and SOD enzymes were determined using the guaiacol (Zelinová et al., 2010) and NBT (Rukmini et al., 2004) methods, respectively. The contents of free sugars, amino acids, and polyphenols were measured using the anthrone (Li and Li, 2013), the ninhydrin (Sun et al., 2006), and the Folin phenol (Li et al., 2008) methods, respectively.

### Loading and Quality Control Sample Preparation

The above-mentioned collected ultra-filtered sap in bigger and outer centrifuge tube (200 μl) and pre-cooled methanol (800 μl) were mixed and centrifuged for 30 min at 4°C at 13,000 *g*; then, the supernatant was divided into four equal parts and lyophilized. The quality control (QC) samples were prepared by mixing aliquots of batch samples to create pooled samples according to the described methods (Sangster et al., 2006; Gika et al., 2007). Briefly, each filtered sap (20 μl) was mixed with pre-cooled methanol of four times the volume and centrifuged for 30 min at 4°C at 13,000 g, and then the supernatant was divided according to the same share allocation with single loading sample preparation and lyophilized. Each lyophilized sample was resolved with 20 μl 20% (v/v) acetonitrile in water and analyzed *via* LC–MS using a Shimadzu LC20AD HPLC system coupled with a Triple TOF 5600 mass spectrometer (AB SCIEX). The order of sample loading was such that four QC samples were randomly assigned at the beginning and two QC samples were randomly allocated between every eight samples, being described in such a way that the QC samples should be randomly analyzed at the beginning and at the end (Sangster et al., 2006; Gika et al., 2007).

The prepared samples were kept at 4°C in the autosampler, and then 2 μl aliquots were taken for analyses using a reverse-phase gradient LC device fitted with a Phenomenex Luna 3 μm C18 column (150 × 2.0 mm) as well as analyses using a hydrophilic interaction chromatography gradient LC fitted with a TSK gel Amide-80 3 μm column (150 × 2.0 mm).

### Reversed-Phase Chromatography Separation

For RPC, we used a binary solvent system delivered as a gradient of 0.1% (v/v) formic acid in water (solvent A) and 0.1% (v/v) formic acid in pure acetonitrile (solvent B) using a flow rate of 300 μl min^–1^, with the column maintained at 45°C. The chromatographic column was equilibrated with 98% solvent A. The starting gradient conditions were 98% solvent A and 2% solvent B. Then, a linear gradient was conducted up to 65% solvent B over 20 min, at which point the solvent composition was increased to 100% solvent B for 5 min. Then, the column was returned to 2% solvent B over the next 1 min and maintained at this level for 4 min, for a total cycle time of 30 min/sample.

### Hydrophilic Interaction Chromatography Separation

For HILIC, we used a binary solvent system delivered as a gradient of 0.1% (v/v) formic acid and 10 mM ammonium formate in water (solvent A) and 0.1% (v/v) formic acid in pure acetonitrile (solvent B) using a flow rate of 200 μl min^–1^, with the column maintained at 45°C. The chromatographic column was equilibrated with 95% solvent B. The starting gradient conditions were 5% solvent A and 95% solvent B. Then, a linear gradient was conducted down to 70% solvent B over 24 min, after which the composition was further decreased to 10% solvent B for 4 min and maintained at this level for 2 min. Then, the solvent composition was again returned to 95% over 1 min and maintained at this level for 4 min, for a total cycle time of 35 min/sample.

### Mass Spectrometer Identification

The compounds separated by LC were analyzed in both positive- and negative-ion modes. The mass spectrometer was operated in the positive and the negative ESI modes with a Duo Spray TM source (AB SCIEX, Canada) connected to an MS/MS (Triple TOF 5600, AB SCIEX). The following parameter settings were used: ion spray voltage, ±5,500 V; ion source temperature, 600°C; curtain gas, 30 psi; both ion source gas 1 and gas 2, 55 psi; TOF-MS scan, m/z 100–1,000 Da of mass range; and scan accumulation time, 0.25 s/spectra. In high sensitivity mode, they were as follows: ion scan m/z range, 50–1,000 Da; ion scan accumulation time, 0.07 s/spectra; declustering potential, ±60 V; and collision energy, ±20 V. In addition, information-dependent acquisition (IDA) was used to acquire MS/MS spectra for ions matching the IDA criteria (excluding isotopes within 4 Da, candidate ions to monitor per cycle: 10).

### Metabolomic Data Processing

MS data (wiff.scan files) were converted into MzXML files using ProteoWizard MSConvert and processed by XCMS for feature detection, retention time correction, and alignment. The metabolites were identified by mass (the errors were less than 25 ppm) and according to MS/MS data that were queried and matched with a laboratory standards database developed by Shanghai Applied Protein Technology Co., Ltd.

In extracted ion features, only variables with more than 50% of the non-zero measurement values in at least one group were retained. Progenesis QI (Non-linear Dynamics) was used for data processing, statistical analyses, and the selection and the identification of significantly different compounds. Specifically, from the resulting chromatograms, mass spectra peaks (compound ions) were aligned (score > 80 as better alignment), picked [relative standard deviation (RSD) among all QC sample runs < 30%] with an m/z and a retention time, and normalized. Among the picked and normalized peaks, significantly different compounds between the LK and the NK groups were screened and selected by partial least squares discriminant analysis (PLS-DA) [variable importance for the projection (VIP) score > 1.0] and volcano plot [*p* < 0.05; fold change (FC) > 2 or <0.5]. Then, the selected compounds were tentatively identified using MetaScope based on neutral mass (m/z tolerance 10 ppm), retention time (for no fragments matching), and fragments (fragment score > 60). Hierarchical clustering analyses were used to evaluate the reasonability of the significantly different compounds between the groups.

Variables (metabolites) that significantly contributed to the clustering and discrimination were identified according to a threshold of VIP values (VIP > 1), which could be generated after PLS-DA processing. To select potential biomarkers worthy of preferential study in the next step, these different metabolites were validated using a *t*-test.

### Statistical Analyses

Three biological replicates were established to quantify cotton seedling growth; the levels of minerals, free sugars, free proteins, malondialdehyde (MDA), polyphenols, and amino acids in sap; the activities of G-POD and SOD in sap; and sap pH. Eight biological replicates were used for quantitative analyses of metabolites in xylem sap for each treatment. Student’s test was used to compare the differences between the LK and the NK treatments.

## Results

### Changes in Seedling Growth, Mineral Contents, and Physiological Traits of Cotton Under K Deficiency

The cotton cultivar DP 99B showed obvious thin plants after 7 days of LK stress (Table 1). Compared to control, some morphological characters of 3-day-old cotton seedlings treated with LK for 7 days were statistically significantly different, including lower dry weights of leaves, stems, and roots and shorter stems and total roots (Table 1). In addition, because there were no significant differences in mean root diameter between the treatments, the differences in root length resulted in significant differences in root surface area and root volume.

**TABLE 1:** Changes in seedling growth of cotton under K deficiency.

Class	Leaf^a^	Stem^a^	Root^a^	Stem length^a^	Total root length^a^	Mean root diameter	Root surface area^a^	Root volume^a^
					
	g dry weight plant^–1^			cm plant^–1^		mm plant^–1^	cm^2^ plant^–1^	cm^3^ plant^–1^
LK	0.13 ± 0.010	0.05 ± 0.006	0.04 ± 0.003	18.96 ± 4.03	511.21 ± 54.36	0.38 ± 0.01	62.73 ± 8.29	0.62 ± 0.11
NK	0.20 ± 0.002	0.08 ± 0.002	0.06 ± 0.001	22.96 ± 0.79	782.81 ± 28.95	0.39 ± 0.03	93.34 ± 0.41	0.89 ± 0.03

*LK, low K; NK, normal K. ^*a*^There was a significant difference between LK and NK treatments according to t-test, p < 0.05, r = 3.*

Compared to controls, the LK group had lower pH, levels of five mineral nutrients (K, Na, Mg, Fe, and Zn), free sugars, free proteins, and polyphenols, and activities of G-POD and SOD but had significantly higher xylem sap volume and Ca^2+^, MDA, and amino acid levels (Tables 2, 3).

**TABLE 2:** Changes in pH and mineral nutrient contents of cotton xylem sap under K deficiency.

Class	pH^a^	Xylem sap volume^a^	K^a^	Na^a^	Ca^a^	Mg^a^	Fe^a^	Zn^a^
			
		ml plant^–1^	mg L^–1^ xylem sap					
LK	5.41 ± 0.06	229.53 ± 31.65	157.39 ± 9.67	184.46 ± 15.98	988.00 ± 51.28	412.49 ± 14.83	7.61 ± 1.34	6.44 ± 1.48
NK	5.97 ± 0.09	181.53 ± 37.09	240.49 ± 43.47	224.96 ± 22.30	830.15 ± 80.74	457.25 ± 31.73	14.08 ± 4.56	13.09 ± 3.76

*LK, low K; NK, normal K. ^*a*^There was a significant difference between LK and NK treatments according to t-test, p < 0.05, r = 3.*

**TABLE 3:** Changes in the physiological characteristics of cotton xylem sap under K deficiency.

Class	MDA^a^	G-POD activity^a^	SOD activity^a^	Free sugar^a^	Free protein^a^	Polyphenol^a^	Amino acid^a^
			
	nmol ml^–1^	U ml^–1^ xylem sap		μ g ml^–1^			
LK	48.24 ± 11.20	54.00 ± 11.63	4.79 ± 0.57	12.25 ± 1.65	2.06 ± 0.13	37.71 ± 1.27	72.06 ± 5.20
NK	43.51 ± 19.68	82.15 ± 13.84	5.60 ± 0.19	16.16 ± 3.78	3.03 ± 0.41	41.93 ± 1.83	53.05 ± 8.02

*LK, low K; NK, normal K. ^*a*^There was a significant difference between LK and NK treatments according to t-test, p < 0.05, r = 3.*

### Analyses of Reproducibility of HPLC–MS/MS

**FIGURE 1:**
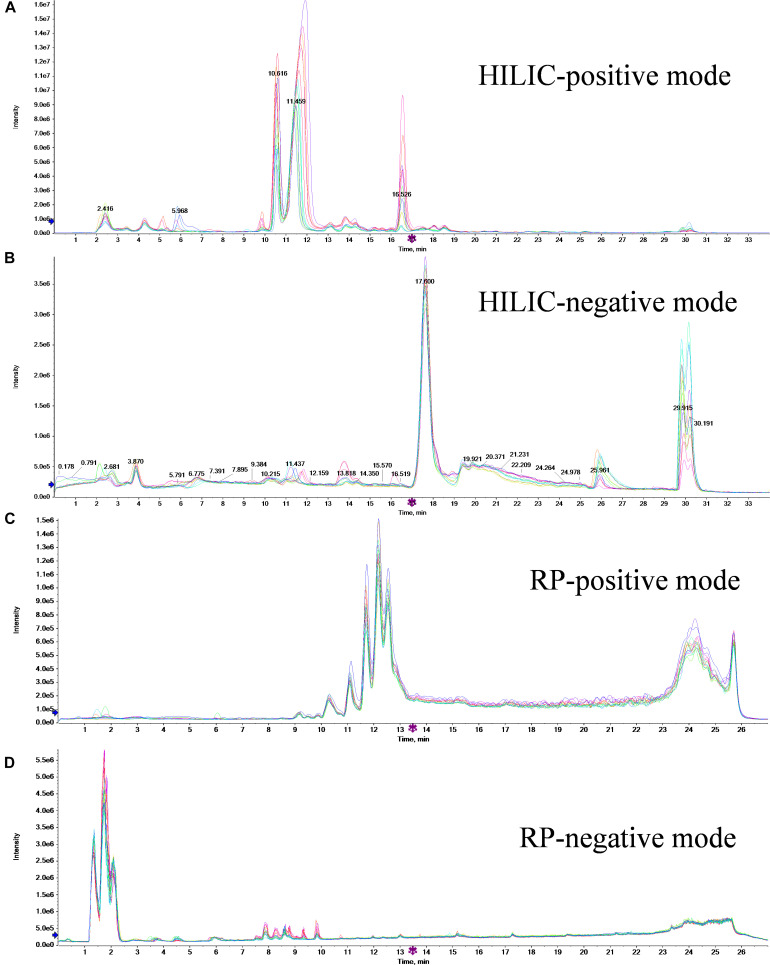
Total ion current chromatograms of 16 samples of two treatments for HILIC-positive **(A)**, HILIC-negative **(B)**, RPC-positive **(C)**, and RPC-negative **(D)** modes.

Metabolites of 16 samples (eight different biological samples from each treatment) and six QC samples were analyzed by HPLC–MS/MS. The HILIC-positive, HILIC-negative, RPC-positive, and RPC-negative total ion current chromatograms obtained from these analyses are shown in Figure 1. The retention peaks of HILIC and RPC mode were detected at around 30.5 and 25.8 min, respectively; therefore, the retention time was set to 35 and 30 min for HILIC and RPC mode analyses, respectively.

The reproducibility of both peak intensity and retention time in each mode is very important when attempting to explain the differences among samples. As shown in Figure 1, the retention time and the peak intensity of all samples in each mode were similar, indicating a high degree of reproducibility of our mass spectrometry data.

### Metabolite Profiles

There were 8,188, 4,820, 2,442, and 5,972 detected peaks in the HILIC-positive, HILIC-negative, RPC-positive, and RPC-negative modes, respectively (Table 4). The numbers of compounds in samples with peak strength RSD ≤ 30% were 642, 186, 329, and 565 in these four modes, respectively.

**TABLE 4:** Significantly different compounds screened and selected by combining partial least squares discriminant analysis and volcano plot under different separation and analysis modes.

Items	Hydrophilic interaction liquid chromatographic separation		Reverse-phase chromatography separation	
	Positive	Negative	Positive	Negative
Detected peaks	8,188	4,820	2,442	5,972
Peaks(relative standard deviation < 30%)	642	186	329	565
Significantly different compounds	56	3	0	23

**FIGURE 2:**
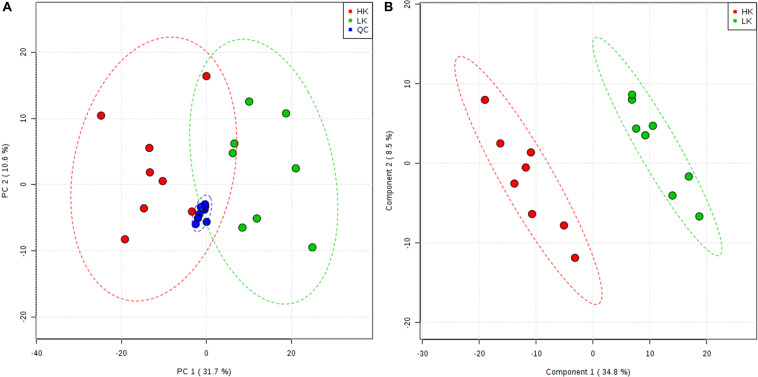
Principal component analysis score plot **(A)** and partial least squares discriminant analysis score plot **(B)** from the HILIC-POS mode.

After the data were cleaned and normalized, a principal component analysis (PCA) of all samples was used to assess the experimental quality. Taking the HILIC-positive ion mode as an example, the PCA score plot (Figure 2A) pooled the QC samples together, indicating that the HPLC–MS/MS process met the required qualifications. However, it was difficult to discriminate between the two treatments in the unsupervised multivariate analyses (Figure 2A). Thus, supervised multivariate analyses using PLS-DA were conducted, which clustered the eight biological replicates of each treatment group, verifying the experimental design and robustness of the method. Two plots of PCA and PLS-DA score were drawn with the top two principal components. The largest variation was represented by principal component 1, which accounted for 31.7% of the PCA score plot (Figure 2A) and 34.8% of the PLS-DA score plot (Figure 2B). The PLS-DA model clearly distinguished the two treatments based on the LC–MS/MS data.

**FIGURE 3:**
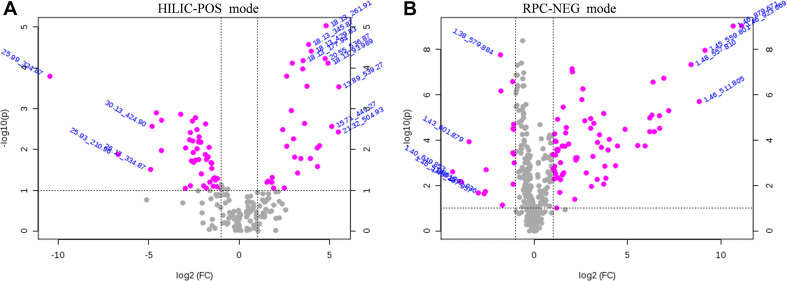
Volcano plots of HILIC-POS **(A)** and RPC-NEG **(B)** showing metabolomic data. The pink dots indicate points-of-interest that display both large-magnitude fold changes (x-axis) and high statistical significance [−log_10_(*p*), y-axis]. The dashed line shows where *p* = 0.05, with points above the line at *p* < 0.05 and points below the line at *p* > 0.05. Points at a fold change less than 2 (log_2_^2^ = 1) and *p* > 0.05 (-log_10_^0^.^05^ = 1.3) are shown in gray.

The VIP score reflects the importance of the variables in the PLS-DA model and was applied to measure the influence of the expression intensity of metabolites on the classification and the interpretation ability of samples of each group, thus helping to screen important metabolites (usually VIP score > 1.0 as screening criteria). Based on the PLS-DA analyses, the critical *p*-value was set to 0.05 for significantly different variables with FC > 2.0. Following the criteria above, 82 significantly different endogenous metabolites (56 in HILIC-POS mode, three in HILIC-NEG, 23 in RPC-NEG mode, and 0 in RPC-POS) between the NK and the LK treatments were screened by volcano plots (Figure 3) for further study.

### Cluster Analyses of Altered Metabolites Under LK Stress

Three heatmaps were generated as graphical representations of the up- and down-regulated expressions of metabolites. Among the 82 metabolites, 38 in the LK treatment showed 2-fold higher upregulation (red color) vs. controls, while 44 showed less than 0.5-fold downregulation (blue color).

**FIGURE 4:**
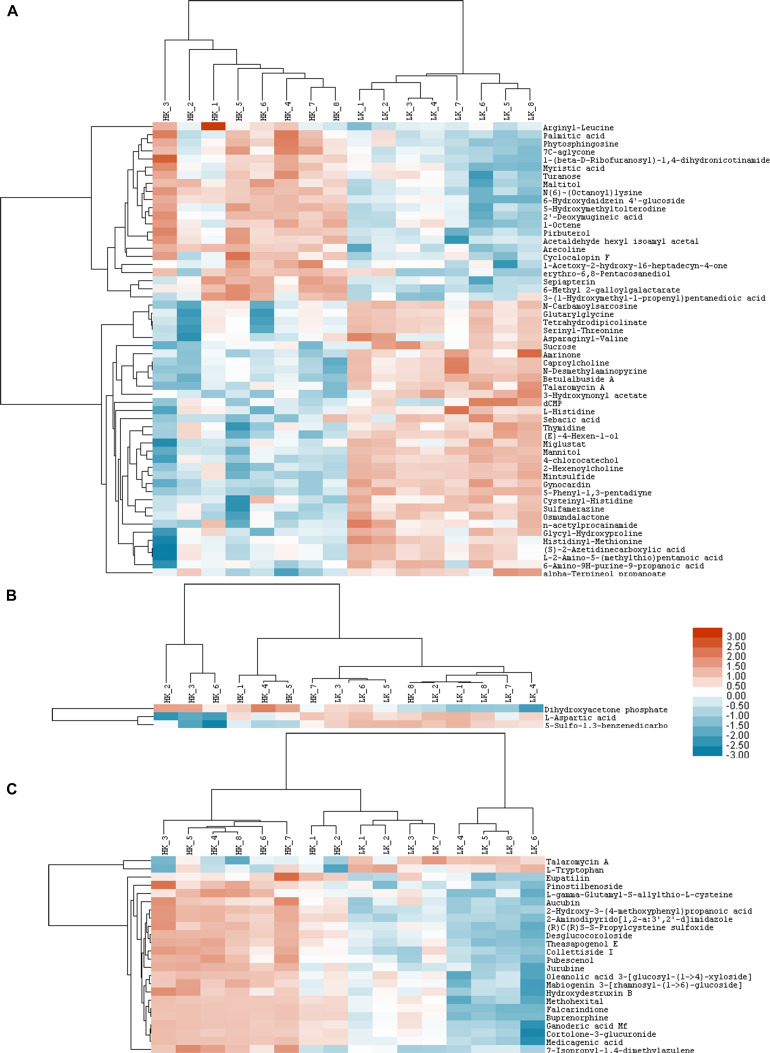
Heat maps of HILIC-POS **(A)**, HILIC-NEG **(B)**, and RPC-NEG **(C)** modes.

More specifically, 34, two, and two metabolites were up-regulated in the HILIC-POS (Figure 4A), HILIC-NEG (Figure 4B), and RPC-NEG (Figure 4C) modes, respectively, with glycerophosphocholine showing the highest increase in upregulation (FC = 29.00) (Table 5). Because heatmaps showed stable repeatability among different samples in the same treatment, 22, one, and 22 down-regulated metabolites could also be easily found in the blue area, i.e., in HILIC-POS (Figure 4A), HILIC-NEG (Figure 4B), and RPC-NEG (Figure 4C) modes, respectively. For example, 6-hydroxydaidzein 4′-glucoside with FC = 0.02 was in the end among the down-regulated metabolites (Table 5).

**TABLE 5:** Comparison of metabolites in the xylem sap of cotton seedlings between low K (LK) and normal K (NK) treatments.

Metabolites	Class	Subclass	FC (LK/NK)	Mode	Compound ID	Adducts	Formula	*P*-value	Variable importance for the projection score
Glycerophosphocholine	PM	Lipid	29.00	HILIC-POS	28.17_258.1093m/z	M + H	C_8_H_20_NO_6_P	0.0070	1.2025
Tetrahydrodipicolinate	PM	α-Amino acid	16.72	HILIC-POS	23.38_189.0863m/z	M + NH_4_	C_7_H_9_NO_4_	0.0087	1.1777
Gynocardin	SM	Other	14.72	HILIC-POS	22.44_304.1004m/z	M + H	C_12_H_17_NO_8_	0.0000	1.6790
Betulalbuside A	PM	Lipid	13.77	HILIC-POS	3.76_333.1917m/z	M + H	C_16_H_28_O_7_	0.0000	1.6347
Glutarylglycine	PM	α-Amino acid	12.43	HILIC-POS	23.41_207.0969m/z	M + NH_4_	C_7_H_11_NO_5_	0.0213	1.0624
Mintsulfide	SM	Terpenoid	12.29	HILIC-POS	3.09_237.1694m/z	M + H	C_15_H_24_S	0.0000	1.6686
2-Hexenoylcholine	PM	Lipid	8.68	HILIC-POS	3.40_223.1540m/z	M + Na	C_11_H_22_NO_2_	0.0000	1.6083
Methyl 4-chloro-1H-indole-3-acetate	SM	Alkaloid	7.47	HILIC-POS	9.59_265.0738m/z	M + ACN + H	C_11_H_10_ClNO_2_	0.0001	1.5174
(E)-4-Hexen-1-ol	PM	Lipid	6.14	HILIC-POS	16.28_118.1227m/z	M + NH_4_	C_6_H_12_O	0.0002	1.5019
Serinyl-threonine	PM	α-Amino acid	5.97	HILIC-POS	23.33_229.0788m/z	M + Na	C_7_H_14_N_2_O_5_	0.0038	1.2671
N-Carbamoylsarcosine	PM	α-Amino acid	5.39	HILIC-POS	25.29_132.0529n	M + H, M + Na	C_4_H_8_N_2_O_3_	0.0026	1.3025
5-Phenyl-1,3-pentadiyne	SM	Other	5.36	HILIC-POS	4.78_158.0963m/z	M + NH_4_	C_11_H_8_	0.0000	1.7931
N-Desmethylaminopyrine	SM	Alkaloid	5.20	HILIC-POS	3.45_259.1539m/z	M + ACN + H	C_12_H_15_N_3_O	0.0000	1.5733
Histidinyl-methionine	PM	α-Amino acid	4.41	HILIC-POS	29.54_328.1425m/z	M + ACN + H	C_11_H_18_N_4_O_3_S	0.0084	1.1826
Caproylcholine	PM	Lipid	4.11	HILIC-POS	3.44_241.1434m/z	M + K	C_11_H_24_NO_2_	0.0001	1.5411
L-Aspartic acid	PM	α-Amino acid	4.04	HILIC-NEG	25.22_114.0192m/z	M -H_2_O - H	C_4_H_7_NO_4_	0.0260	1.8390
Osmundalactone	SM	Phenol	3.95	HILIC-POS	9.26_129.0543m/z	M + H	C_6_H_8_O_3_	0.0002	1.4901
L-Histidine	PM	α-Amino acid	3.77	HILIC-POS	29.05_156.0764m/z	M + H	C_6_H_9_N_3_O_2_	0.0046	1.2474
6-Amino-9H-purine-9-propanoic acid	PM	Nucleotide	3.43	HILIC-POS	24.32_230.0628m/z	M + Na	C_8_H_9_N_5_O_2_	0.0089	1.1749
4-Chlorocatechol	SM	Phenol	2.98	HILIC-POS	29.88_182.9617m/z	M + K	C_6_H_5_ClO_2_	0.0000	1.5644
Glycyl-hydroxyproline	PM	α-Amino acid	2.90	HILIC-POS	25.71_230.1130m/z	M + ACN + H	C_7_H_12_N_2_O_4_	0.0056	1.2269
dCMP	PM	Nucleotide	2.78	HILIC-POS	19.35_325.0894m/z	M + NH_4_	C_9_H_14_N_3_O_7_P	0.0277	1.0240
Asparaginyl-valine	PM	α-Amino acid	2.75	HILIC-POS	29.14_249.1559m/z	M + NH_4_	C_9_H_17_N_3_O_4_	0.0269	1.0283
n-Acetylprocainamide	SM	Phenol	2.63	HILIC-POS	3.02_295.2124m/z	M + NH_4_	C_15_H_23_N_3_O_2_	0.0067	1.2082
5-Sulfo-1,3-benzenedicarboxylic acid	SM	Phenol	2.61	HILIC-NEG	29.86_245.9819n	M -H_2_O - H, M - H	C_8_H_6_O_7_S	0.0040	2.2210
Propyl propane thiosulfonate	SM	Other	2.55	HILIC-POS	3.76_229.1072m/z	M + H	C_11_H_16_O_5_	0.0002	1.4824
Talaromycin A	SM	Other	2.51	HILIC-POS	3.68_272.1859m/z	M + ACN + H	C_12_H_22_O_4_	0.0004	1.4473
Cysteinyl-histidine	PM	α-Amino acid	2.49	HILIC-POS	5.02_281.0676m/z	M + Na	C_9_H_14_N_4_O_3_S	0.0096	1.1662
Sucrose	PM	Carbohydrate	2.43	HILIC-POS	25.41_365.1061m/z	M + Na	C_12_H_22_O_11_	0.0023	1.3152
(S)-2-Azetidinecarboxylic acid	PM	α-Amino acid	2.37	HILIC-POS	29.22_102.0551m/z	M + H	C_4_H_7_NO_2_	0.0087	1.1786
L-2-Amino-5-(methylthio)pentanoic acid	PM	α-Amino acid	2.37	HILIC-POS	29.22_164.0732m/z	M + H	C_6_H_13_NO_2_S	0.0113	1.1470
Talaromycin A	SM	Other	2.23	RPC-NEG	12.33_275.1491m/z	M + FA –H	C_12_H_22_O_4_	0.0000	1.3110
Thymidine	PM	Nucleotide	2.22	HILIC-POS	16.27_281.0513m/z	M + K	C_10_H_14_N_2_O_5_	0.0056	1.2268
Mannitol	PM	Carbohydrate	2.20	HILIC-POS	19.64_205.0679m/z	M + Na	C_6_H_14_O_6_	0.0000	1.6456
L-Tryptophan	PM	α-Amino acid	2.20	RPC-NEG	5.87_203.0820m/z	M – H	C_11_H_12_N_2_O_2_	0.0090	1.0610
alpha-Terpineol propanoate	SM	Terpenoid	2.16	HILIC-POS	16.27_228.1957m/z	M + NH_4_	C_13_H_22_O_2_	0.0023	1.3141
Sebacic acid	PM	Lipid	2.04	HILIC-POS	4.13_244.1545m/z	M + ACN + H	C_10_H_18_O_4_	0.0014	1.3593
3-Hydroxynonyl acetate	PM	Lipid	2.04	HILIC-POS	8.92_244.1915m/z	M + ACN + H	C_11_H_22_O_3_	0.0002	1.4997
Aucubin	SM	Terpenoid	0.50	RPC-NEG	7.15_383.0721m/z	M + K -2H	C_15_H_22_O_9_	0.0000	1.3010
1-(beta-D-Ribofuranosyl)-1,4-dihydronicotinamide	SM	Alkaloid	0.48	HILIC-POS	30.04_279.0942m/z	M + Na	C_11_H_16_N_2_O_5_	0.0010	1.3842
Turanose	PM	Carbohydrate	0.48	HILIC-POS	22.50_360.1512m/z	M + NH_4_	C_12_H_22_O_11_	0.0188	1.0796
7-Isopropyl-1,4-dimethylazulene	SM	Terpenoid	0.48	RPC-NEG	8.66_441.2811m/z	2M + FA - H	C_15_H_18_	0.0050	1.1140
Acetaldehyde hexyl isoamyl acetal	PM	Lipid	0.47	HILIC-POS	9.18_255.1710m/z	M + K	C_13_H_28_O_2_	0.0004	1.4470
Hydroxydestruxin B	PM	α-amino acid	0.47	RPC-NEG	7.87_654.3665m/z	M + FA – H	C_30_H_51_N_5_O_8_	0.0020	1.1840
Arecoline	SM	Alkaloid	0.47	HILIC-POS	3.24_156.1022m/z	M + H	C_8_H_13_NO_2_	0.0001	1.5429
Collettiside I	SM	Terpenoid	0.46	RPC-NEG	8.16_613.3155m/z	M + K - 2H	C_33_H_52_O_8_	0.0000	1.3730
Cyclocalopin F	SM	Phenol	0.46	HILIC-POS	3.28_317.0976m/z	M + Na	C_15_H_18_O_6_	0.0045	1.2504
(R)C(R)S-S-Propylcysteine sulfoxide	PM	α-Amino acid	0.46	RPC-NEG	1.85_200.0379m/z	M + Na - 2H	C_6_H_13_NO_3_S	0.0000	1.3730
Mabiogenin 3-[rhamnosyl-(1- > 6)-glucoside]	SM	Phenol	0.45	RPC-NEG	7.28_817.4400m/z	M + Na - 2H	C_42_H_68_O_14_	0.0030	1.1630
Jurubine	SM	Terpenoid	0.45	RPC-NEG	8.76_630.3815m/z	M + Cl	C_33_H_57_NO_8_	0.0000	1.3710
Neotussilagine	SM	Alkaloid	0.43	HILIC-POS	9.24_241.1557m/z	M + ACN + H	C_10_H_17_NO_3_	0.0001	1.5455
Pubescenol	SM	Terpenoid	0.41	RPC-NEG	8.81_509.2678m/z	M + Cl	C_28_H_42_O_6_	0.0000	1.3310
3-(1-Hydroxymethyl-1-propenyl)pentanedioic acid	PM	Lipid	0.41	HILIC-POS	4.83_220.1178m/z	M + NH_4_	C_9_H_14_O_5_	0.0111	1.1490
2′-Deoxymugineic acid	SM	Other	0.40	HILIC-POS	24.18_304.1245n	M + H, M + Na, M + K	C_12_H_20_N_2_O_7_	0.0002	1.4868
7C-Aglycone	SM	Other	0.40	HILIC-POS	3.89_299.1302m/z	M + H	C_18_H_18_O_4_	0.0006	1.4171
Dihydroxyacetone phosphate	PM	Carbohydrate	0.39	HILIC-NEG	29.21_204.9685m/z	M + Cl	C_3_H_7_O_6_P	0.0070	2.1330
5-Hydroxymethyltolterodine	SM	Phenol	0.38	HILIC-POS	3.93_383.2665m/z	M + ACN + H	C_22_H_31_NO_2_	0.0004	1.4523
L-gamma-Glutamyl-S-allylthio-L-cysteine	PM	α-Amino acid	0.38	RPC-NEG	2.23_359.0125m/z	M + K - 2H	C_11_H_18_N_2_O_5_S_2_	0.0050	1.1070
Pinostilbenoside	SM	Phenol	2.37	RPC-NEG	1.99_441.0985m/z	M + K - 2H	C_21_H_24_O_8_	0.0010	1.2480
Oleanolic acid 3-[glucosyl-(1- > 4)-xyloside]	SM	Terpenoid	0.35	RPC-NEG	7.23_771.4352m/z	M + Na - 2H	C_41_H_66_O_12_	0.0030	1.1530
Sepiapterin	PM	Nucleotide	0.34	HILIC-POS	4.77_260.0755m/z	M + Na	C_9_H_11_N_5_O_3_	0.0010	1.3840
Eupatilin	SM	Phenol	0.33	RPC-NEG	12.65_365.0615m/z	M + Na - 2H	C_18_H_16_O_7_	0.0030	1.1700
Theasapogenol E	SM	Terpenoid	0.31	RPC-NEG	8.29_541.2934m/z	M + K - 2H	C_30_H_48_O_6_	0.0000	1.4320
1-Acetoxy-2-hydroxy-16-heptadecyn-4-one	PM	Lipid	0.29	HILIC-POS	9.86_342.2639m/z	M + NH_4_	C_19_H_32_O_4_	0.0192	1.0771
Maltitol	PM	Carbohydrate	0.27	HILIC-POS	19.59_345.1390m/z	M + H	C_12_H_24_O_11_	0.0001	1.5399
Palmitic acid	PM	Lipid	0.25	HILIC-POS	8.70_274.2743m/z	M + NH_4_	C_16_H_32_O_2_	0.0088	1.1764
2-Aminodipyrido[1,2-a:3′,2′-d]imidazole	SM	Alkaloid	0.25	RPC-NEG	2.30_219.0446m/z	M + Cl	C_10_H_8_N_4_	0.0000	1.5770
Phytosphingosine	PM	Lipid	0.24	HILIC-POS	8.37_318.3015m/z	M + H	C_18_H_39_NO_3_	0.0011	1.3781
2-Hydroxy-3-(4-methoxyphenyl)propanoic acid	SM	Phenol	0.24	RPC-NEG	2.27_217.0473m/z	M + Na - 2H	C_10_H_12_O_4_	0.0000	1.5730
Arginyl-leucine	PM	α-Amino acid	0.23	HILIC-POS	27.98_288.2036m/z	M + H	C_12_H_25_N_5_O_3_	0.0227	1.0535
Ganoderic acid Mf	SM	Terpenoid	0.22	RPC-NEG	8.31_533.3278m/z	M + Na - 2H	C_32_H_48_O_5_	0.0010	1.2800
1-Octene	SM	Other	0.21	HILIC-POS	8.78_130.1590m/z	M + NH_4_	C_8_H_16_	0.0014	1.3547
Myristic acid	PM	Lipid	0.14	HILIC-POS	9.15_246.2430m/z	M + NH_4_	C_14_H_28_O_2_	0.0077	1.1925
Cortolone-3-glucuronide	SM	Terpenoids	0.12	RPC-NEG	8.76_523.2601m/z	M - H_2_O - H	C_27_H_42_O_11_	0.0110	1.0360
6-Methyl 2-galloylgalactarate	PM	Carbohydrate	0.10	HILIC-POS	12.77_394.0974m/z	M + NH_4_	C_14_H_16_O_12_	0.0000	1.6801
L-Phenylalanyl-L-proline	PM	α-Amino acid	0.10	RPC-NEG	8.29_523.2607m/z	2M – H	C_14_H_18_N_2_O_3_	0.0050	1.1090
Medicagenic acid	SM	Terpenoid	0.08	RPC-NEG	8.76_539.2786m/z	M + K - 2H	C_30_H_46_O_6_	0.0000	1.3410
Desglucocoroloside	SM	Terpenoid	0.08	RPC-NEG	8.18_539.2788m/z	M + Cl	C_29_H_44_O_7_	0.0000	1.4800
N(6)-(Octanoyl)lysine	PM	α-Amino acid	0.06	HILIC-POS	28.71_295.1995m/z	M + Na	C_14_H_28_N_2_O_3_	0.0000	1.6709
Falcarindione	SM	Terpenoid	0.05	RPC-NEG	8.33_557.2890m/z	2M + FA - H	C_17_H_20_O_2_	0.0010	1.2260
Erythro-6,8-pentacosanediol	PM	Lipid	0.04	HILIC-POS	8.05_423.3601m/z	M + K	C_25_H_52_O_2_	0.0116	1.1430
6-Hydroxydaidzein 4′-glucoside	SM	Phenol	0.02	HILIC-POS	3.77_455.0921m/z	M + Na	C_21_H_20_O_10_	0.0011	1.3765

*Significantly different metabolites were selected based on partial least squares discriminant analysis and volcano plots, all with p < 0.05; variable importance for the projection scores > 1.0 and FC > 2.0. FC(LK/NK), fold change (LK vs. NK); PM, primary metabolite; SM, secondary metabolite.*

### Changes in Primary and Secondary Metabolites Between the Two Treatments

**FIGURE 5:**
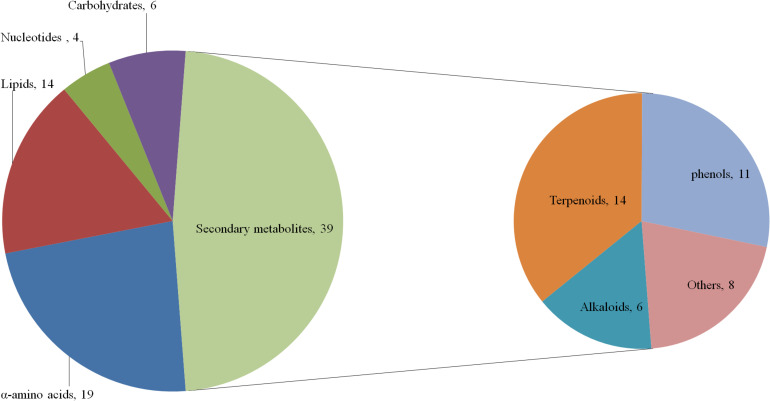
Compound pie chart of primary and secondary metabolites based on 82 significant metabolites.

According to the traditional classification method and referring to the classifications of Kyoto Encyclopedia of Genes and Genomes (KEGG) database and Irchhaiya et al. (2015), 82 different metabolites were classified into 43 primary metabolites and 39 secondary metabolites. These primary metabolites were further divided into 19 α-amino acids (including derivatives), six carbohydrates (including derivatives), 14 lipids (including derivatives), and four nucleotides (including derivatives). The secondary metabolites included 14 terpenoids, 11 phenols, six alkaloids, and 8 “others” (Figure 5).

**FIGURE 6:**
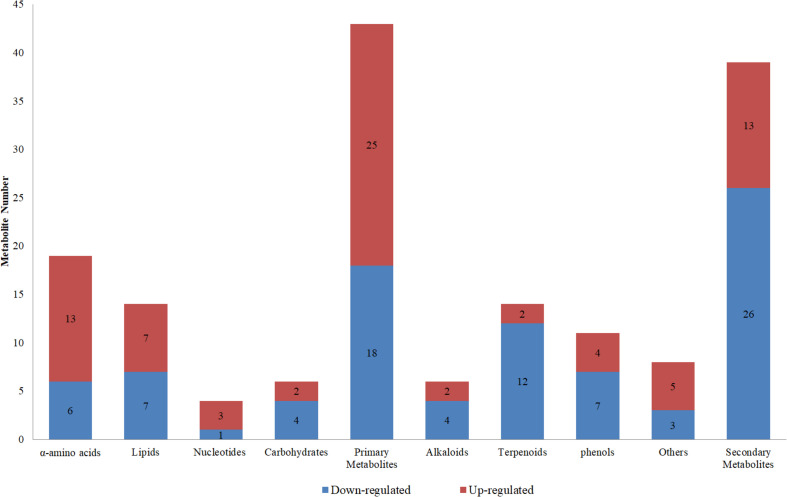
Classifications of up- and down-regulated metabolites in the low-K treatment compared to controls.

Compared to controls, the sub-class ratios of 25 up-regulated and 18 down-regulated primary metabolites in the LK treatment were 13:6 for α-amino acids, 7:7 for lipids, 3:1 for nucleotides, and 2:4 for carbohydrates; those of 13 up-regulated and 26 down-regulated secondary metabolites in the LK treatment were 2:4 for alkaloids, 2:12 for terpenoids, 4:7 for phenols, and 5:3 for others (Figure 6). These results show that LK stress promoted the accumulations of primary metabolites and inhibited the productions of secondary metabolites in the xylem sap of cotton.

### Metabolic Pathway Analyses

**FIGURE 7:**
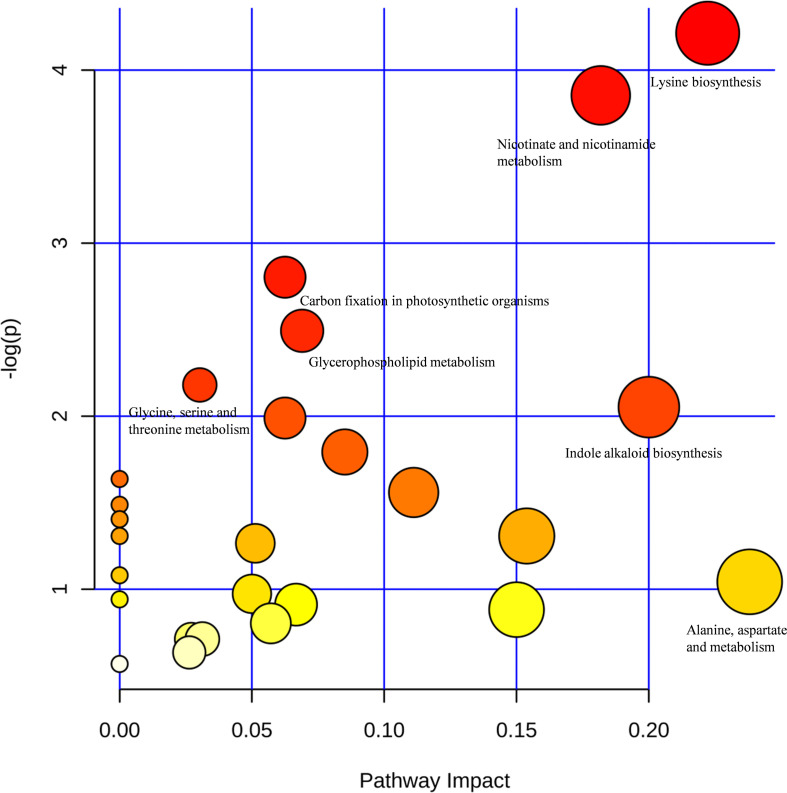
Pathway impact plot of 26 matched Kyoto Encyclopedia of Genes and Genomes (KEGG) pathways: representation of the metabolites matching the KEGG pathways displayed according to their significance by pathway enrichment analyses (y-axis) and impact factors by pathway topology analyses (x-axis). The redder colors indicate lower *P*-values (higher -log_*e*_*P*, *e* ≈ 2.71828), and the larger circles represent higher impact factors. The lower *P*-values and the larger impact factor indicate pathways that were greatly influenced. The pathway impact factor is calculated by the cumulative percentage of the matched metabolites, and the maximum impact factor of each pathway is 1.

The pathway impact plot (Figure 7) provides an overview of 26 metabolites that matched the KEGG pathways using MetaboAnalyst 4.0 online^1^. The results indicate that lysine biosynthesis and nicotinate and nicotinamide metabolism were the mainly pathways involved in the response to LK stress (red circles in the upper-right corner of the graph; more details can be found in Table 6). L-Aspartic acid (FC = 4.04) is an important α-amino acid, and it matched up to 10 pathways including amino acid synthesis and metabolism, nicotinate and nicotinamide metabolism, carbon fixation in photosynthetic organisms, and minoacyl-tRNA biosynthesis. Furthermore, both L-tryptophan (FC = 2.20) and dihydroxyacetone phosphate (FC = 0.39) matched six metabolic pathways.

**TABLE 6:** Overview of the pathway analyses of 26 metabolites matching the Kyoto Encyclopedia of Genes and Genomes pathways.

No.	Pathway name	Total	Hits	Involved metabolites	Impact
1	Alanine, aspartate, and glutamate metabolism	22	1	L-aspartic acid	0.24
2	Lysine biosynthesis	10	2	L-aspartic acid, tetrahydrodipicolinate	0.22
3	Indole alkaloid biosynthesis	7	1	L-tryptophan	0.20
4	Nicotinate and nicotinamide metabolism	12	2	L-Aspartic acid, dihydroxyacetone phosphate	0.18
5	Fructose and mannose metabolism	16	1	Dihydroxyacetone phosphate	0.15
6	Tryptophan metabolism	27	1	L-tryptophan	0.15
7	Beta-alanine metabolism	12	1	L-aspartic acid	0.11
8	Pyrimidine metabolism	38	2	dCMP, thymidine	0.09
9	Glycerophospholipid metabolism	25	2	Glycerophosphocholine, dihydroxyacetone phosphate	0.07
10	Galactose metabolism	26	1	Sucrose	0.07
11	Carbon fixation in photosynthetic organisms	21	2	L-aspartic acid, dihydroxyacetone phosphate	0.06
12	Aminoacyl-tRNA biosynthesis	67	3	L-histidine, L-aspartic acid, L-tryptophan	0.06
13	Starch and sucrose metabolism	30	1	Sucrose	0.06
14	Glucosinolate biosynthesis	54	2	Homomethionine, L-tryptophan	0.05
15	Inositol phosphate metabolism	24	1	Dihydroxyacetone phosphate	0.05
16	Cysteine and methionine metabolism	34	1	L-aspartic acid	0.03
17	Glycine, serine, and threonine metabolism	30	2	L-aspartic acid, L-tryptophan	0.03
18	Fatty acid metabolism	34	1	Palmitic acid	0.03
19	Arginine and proline metabolism	38	1	L-aspartic acid	0.03
20	Cyanoamino acid metabolism	11	1	L-aspartic acid	0.00
21	Fatty acid elongation in mitochondria	13	1	Palmitic acid	0.00
22	Fatty acid biosynthesis	49	2	Myristic acid, palmitic acid	0.00
23	Histidine metabolism	16	1	L-Histidine	0.00
24	Phenylalanine, tyrosine, and tryptophan biosynthesis	21	1	L-Tryptophan	0.00
25	Glycolysis or gluconeogenesis	25	1	Dihydroxyacetone phosphate	0.00
26	Biosynthesis of unsaturated fatty acids	42	1	Palmitic acid	0.00

## Discussion

### Changes in Morphology and Physiology of Cotton Seedlings Under LK Stress

K is an inorganic element necessary for plant growth and development. K deficiency can lead to a decrease in plant metabolic substances such as free protein in the xylem sap, which leads to a thin morphology (Zhang et al., 2015a, b; Fontana et al., 2020). This study confirmed such changes of morphology after 7 days of LK stress (Table 1) and additionally showed the changes in metabolism. Although the volume of xylem sap significantly increased under LK stress, this may have been due to a higher root pressure caused by a decrease in K content (Zhang Z. et al., 2016).

K^+^ is the anti-anion needed for electroneutralization. A decrease in K^+^ concentration in plant tissues and cells can lead to cation imbalance (Armengaud et al., 2009), which in the present study was reflected by the significant decrease in pH value caused by LK treatment (Table 2). Under LK stress, the inorganic ions with positive charge (e.g., Na^+^, Mg^2+^, Fe^2+^, Zn^2+^) decreased in the xylem sap (Table 2), and the decrease in the activities of protective enzymes such as SOD and POD indirectly led to the accumulation of toxic peroxides such as MDA (Table 3). Meanwhile, the concentration of Ca^2+^ as the secondary messenger in plant cells increased (Table 2), indicating that Ca^2+^ could be used as a response signal of cotton seedlings to LK stress (Girón-Calle and JayForman, 2000; Guo et al., 2013). To maintain charge balance under LK stress, plants enhance positively charged amino acids and inhibit negatively charged amino acids; these are strategies for plants to maintain charge balance under LK stress (Zeng et al., 2018). In this study, we observed that the contents of amino acid levels in the xylem sap significantly increased under LK stress (pH = 5.41), and the FCs of positively charged L-histidine (pI = 7.59) and L-tryptophan (pI = 5.89) were higher than that of negatively charged L-aspartic acid (pI = 2.97) (Table 5), which may have been caused by the requirement of charge balance.

### Changes in the Metabolome of Cotton Xylem Sap Under LK Stress

The metabolome reflects the life activities that are happening at a certain moment, and it can directly reflect the impact of environmental changes on organisms (Dixon et al., 2002). The metabolites of eight different biological samples were analyzed by HPLC–MS/MS under each treatment, and 82 metabolites with significant differences between the treatments were screened by volcano plots (Figure 3 and Table 5).

#### Changes in Primary Metabolites of Cotton Xylem Sap Under LK Stress

Carbohydrate metabolism plays a key role in primary metabolism in plants, provides energy for normal growth and development, and acts as a bridge in protein, fat, and nucleic acid metabolism (Rolland et al., 2006). Compared to controls, there were two significantly up-regulated carbohydrates (sucrose and mannitol) in the xylem sap of LK plants (Table 5). Sucrose can act as an osmotic regulator to generate osmotic pressure or as a substrate to produce energy, and it can also induce resistance to adverse conditions through signal transduction (Smeekens, 2000; Hanson et al., 2007). Mannitol is a sugar alcohol with six hydroxyl groups formed after the reduction of mannose. Sugar alcohols can improve the stress resistance of plants by regulating cell permeability and acting as free-radical scavengers (Tarczynski et al., 1993; Stoop et al., 1996; Geng et al., 2011); when they form complexes with nutrient elements such as calcium, they play an important role in vegetable and fruit production (Ding et al., 2015; Li et al., 2018). Mannitol levels are significantly increased under high-temperature stress in *Poa pratensis* (Du et al., 2011) and drought stress in *Fraxinus excelsior* xylem (Patonnier et al., 1999). Similarly, in our study, the sucrose and the mannitol levels in the xylem sap of cotton seedlings were significantly increased under LK stress, which might have been an adaptive change under K deficiency.

Besides carbohydrates, the levels of several other primary metabolites changed significantly under LK stress, including 19 amino acids, 14 lipids, and four nucleic acids (Figure 6). These metabolites are involved in tricarboxylic acid cycle, glycolysis, phospholipid/fatty acid synthesis, nitrogen assimilation, and shikimic acid pathway (Table 6), which are precursors of secondary metabolism. Among them, the levels of some metabolites found in cell membrane including three cholines (glycerophosphatecholine, 2-hexenylcholine, and caproylcholine) and desglucocoroloside and a variety of α-amino acids and their derivatives (dipeptides, such as arginyl-leucine, asparaginyl-valine, cysteinyl-histidine, glycyl-hydroxyproline, histidinyl-methionine, and serinyl-threonine) were significantly increased under LK stress (Tables 5, 6), indicating that protein was metabolized abnormally, resulting in damage to cell membranes.

It is noteworthy that glycerophosphocholine is the major glycerophospholipid in eukaryotic cellular membranes (>50% of all phospholipids) where it makes up the bulk of the bilayer with other lipid classes (e.g., cholesterol) dispersed for fluidity. Glycerophosphocholine is also an important precursor for other lipids, such as sphingomyelin or choline plasmalogens as well as the second messengers diacylglycerol, (lyso-)phosphatidic acid, and arachidonic acid (Triebl, 2016). The dramatic upregulation of glycerophosphocholine under LK stress suggests that it can act as an important signal for root–shoot communication like acetylcholine (Wang et al., 2003).

#### Changes in Secondary Metabolites of Cotton Xylem Sap Under LK Stress

Generally, the well-known anti-abiotic stress metabolites include proline, free sugars, free proteins, and antioxidant enzymes (Han et al., 2013; Guo et al., 2018). However, these resistance markers almost are secondary metabolites produced by plants that help fight against various stresses in nature (Tiwari and Rana, 2015). At present, there are more than 2,140,000 known secondary metabolites produced in the plant kingdom (Thirumurugan et al., 2018). Plants generally open a series of secondary metabolic pathways under stress, among which the phenylalanine metabolic pathway that produces flavonoids, lignin, alkaloids, and other resistant substances is the most important (Dixon et al., 2002).

A total of 26 secondary metabolites belonging to phenols, terpenoids, alkaloids, and other categories significantly decreased under LK stress, including 12 terpenoids (Figure 6). Terpenoids derived from carbon metabolism are volatile (Wu et al., 2017), and their significant down-regulation under LK stress reduces the loss of carbon source metabolites to maintain basic growth needs. Among them, desglucocoroloside, which is polycyclic triterpenoid, is not only an antioxidant (Bosak et al., 2008) but also a membrane stabilizer (Wishart et al., 2017), and its significant downregulation (Table 5) may reflect a weaker antioxidant capacity under LK stress compared to controls. Furthermore, 6-hydroxydaidzein 4′-glucoside, an O-glycosylated derivative of isoflavonoids, which is a natural product derived from 3-phenylchromen-4-one, was the most down-regulated secondary metabolite (Table 5), although there has been no report on the physiological effects of 6-hydroxydaidzein 4′-glucoside, its synthetic precursors, and isoflavonoid properties, indicating that it has an antioxidant function (Castellano and Torrens, 2015).

In addition, 13 secondary metabolites were significantly up-regulated under LK stress (Figure 6). Among them, gynocardin and mintsulfide were up-regulated by 14.72 and 12.29 times, respectively, compared to NK (Table 5). Gynocardin is the first cyclopentenoid glycoside discovered in plants, and its chemical structure has been determined (Webber and Miller, 2008), and cyclopentenone is the glycoside ligand of gynocardin, containing a cyanide group toxic to plants, which might be one of the reasons why the total length, surface area, and volume of roots significantly decreased under LK stress (Siegieñ and Bogatek, 2006). In *Pangium edule* Reinw, soil condition affects the content of gynocardin (Yuningsih, 2008). Mintsulfide, as a volatile metabolite that consumes nutrients, is an enol ester of sulfur-containing sesquiterpene, which might be a signal substance produced by cotton seedlings in response to LK stress (Drahl, 2014). However, because some of the secondary metabolites detected were newly discovered from cotton, their structures, roles, and functions need to be further identified.

### Metabolic Pathway of Cotton Xylem Sap Under LK Stress

The metabolic pathway analyses indicated that 10 principal pathways of the metabolites in response to LK stress involved L-aspartic acid, including lysine biosynthesis and nicotinate and nicotinamide metabolism (Table 6). Tetrahydrodipicolinate (THDP) and L-aspartic acid were strongly up-regulated and are involved in lysine biosynthesis. L-aspartic acid was synthesized by the reaction of L-aspartic acid to diaminopimelate, and then THDP was created to synthesize lysine (Griffin et al., 2012). Lysine is not only an essential component of all proteins but also an important signal amino acid, which plays an important part in regulating plant growth and the response to the environment (Galili et al., 2001; Zhang X. et al., 2016). Furthermore, the inhibition pathway of lysine biosynthesis provides an attractive target for the development of new herbicides. In the most primitive pathway of nicotinate biosynthesis, quinolinic acid, which is a precursor of NAD^+^, is synthesized from L-aspartic acid and dihydroxyacetone phosphate (Cleaves and Miller, 2001), and the latter was significantly down-regulated under LK stress. In addition, L-aspartic acid is involved in the metabolism of alanine, aspartate, and glutamate, and L-tryptophan is involved in the biosynthesis of indole alkaloids; all of these are also worthy of attention.

## Conclusion

K deficiency significantly altered ion uptake and organic substance metabolism and further caused changes in the physiology and the morphology levels in cotton. In detail, K deficiency disturbed cation absorption that caused acidity in the xylem sap. K deficiency also reduced the antioxidant capacity of cotton cells and further resulted in membrane damage that altered the primary and the secondary metabolism in cells, including decreased content of free sugar and soluble proteins as well as increased content of amino acids. Our results also show that cotton plants could positively adapt to K deficiency, which was evidenced by several observed phenomena that included the following: (1) more sucrose and mannitol were generated to balance the osmosis pressure caused by the K deficiency, (2) the reduction of volatile terpenoids reduced carbon loss to maintain basic growth, (3) compared with sufficient potassium, the glycerophosphocholine in the xylem sap was increased by nearly 29-folds, indicating that the roots coordinated the growth of leaves and roots under K deficiency.

## Data Availability Statement

The original contributions presented in the study are included in the article/supplementary material, further inquiries can be directed to the corresponding author/s.

## Author Contributions

XZ, GW, and ZZ designed the study and wrote the manuscript. XZ analyzed xylem sap metabolites quantification, classification, and related pathways. GW, HX, JZ, QW, and ZZ cultivated cotton seedlings, collected xylem sap, and determined the morphological indexes of cotton seedlings and the physiological indicators of xylem sap. BZ improved the manuscript. All co-authors finally approved the manuscript for submission.

## Conflict of Interest

The authors declare that the research was conducted in the absence of any commercial or financial relationships that could be construed as a potential conflict of interest.

## Footnotes

1 www.metaboanalyst.ca
